# An evaluation of the potential for drug–drug interactions between bendamustine and rituximab in indolent non-Hodgkin lymphoma and mantle cell lymphoma

**DOI:** 10.1007/s00280-014-2445-5

**Published:** 2014-03-28

**Authors:** Mona Darwish, John M. Burke, Edward Hellriegel, Philmore Robertson, Luann Phillips, Elizabeth Ludwig, Mihaela C. Munteanu, Mary Bond

**Affiliations:** 1 Malvern, PA USA; 2 Teva Branded Pharmaceutical Products R&D, Inc., 41 Moores Road, Frazer, PA 19355 USA; 3 Rocky Mountain Cancer Centers, 1700 S. Potomac Street, Aurora, CO 80012 USA; 4 US Oncology Research, 10101 Woodloch Forest Drive, The Woodlands, TX 77380 USA; 5 Global Nonclinical DMPK, Teva Branded Pharmaceutical Products R&D, Inc., 145 Brandywine Parkway, West Chester, PA 19380 USA; 6 Cognigen Corporation, 395 South Youngs Road, Buffalo, NY 14221 USA

**Keywords:** Bendamustine, Rituximab, Drug–drug interaction, Systemic exposure, Non-Hodgkin lymphoma

## Abstract

**Purpose:**

Bendamustine plus rituximab has been reported to be effective in treating lymphoid malignancies. This analysis investigated the potential for drug–drug interactions between the drugs in patients with indolent non-Hodgkin lymphoma or mantle cell lymphoma.

**Methods:**

Data were derived from a bendamustine–rituximab combination therapy study, a bendamustine monotherapy study, and published literature on rituximab monotherapy and combination therapy. Analysis of the potential for rituximab to affect bendamustine systemic exposure included comparing bendamustine concentration–time profile following monotherapy to that following combination therapy and comparing model-predicted Bayesian bendamustine clearance in the presence and absence of rituximab. Analysis of the potential for bendamustine to affect rituximab systemic exposure included plotting observed minimum, median, and maximum serum rituximab concentrations at the end of rituximab infusion (EOI) and 24 h and 7 days post-infusion in patients receiving combination therapy versus concentrations reported in literature following rituximab monotherapy.

**Results:**

The established population pharmacokinetic model following bendamustine monotherapy was evaluated to determine its applicability to combination therapy for the purpose of confirming lack of pharmacokinetic interaction. The model adequately described the bendamustine concentration–time profile following monotherapy and combination therapy in adults. There was no statistically significant difference in estimated bendamustine clearance either alone or in combination. Also, rituximab concentrations from EOI to 24 h and 7 days demonstrated a pattern of decline similar to that seen in rituximab studies without bendamustine, suggesting that bendamustine does not affect the rituximab clearance rate.

**Conclusions:**

Neither bendamustine nor rituximab appears to affect systemic exposure of the other drug when coadministered.

## Introduction

Bendamustine is a novel alkylating agent indicated for the treatment of chronic lymphocytic leukemia (CLL) and indolent B cell non-Hodgkin lymphoma (NHL) that has progressed during or within 6 months of treatment with rituximab or a rituximab-containing regimen [1–4]. Rituximab, a chimeric murine/human monoclonal IgG_1_ kappa antibody directed against the CD20 antigen, is indicated for the treatment of CLL and NHL [5–8]. Small molecule drugs, such as bendamustine, are increasingly being used in combination with biologics to treat various diseases [9–12], and such combinations may be especially beneficial in patients with lymphoid malignancies [13]. Indeed, data from several clinical trials have shown that bendamustine plus rituximab is an effective therapy in indolent NHL, with overall response rates ranging from 69–93 % [3, 14–16].

The mechanisms of action of bendamustine and rituximab are substantially different, and the two drugs combined have the potential to act synergistically to induce apoptosis [17]. Bendamustine is a bifunctional mechlorethamine derivative containing a purine-like benzimidazole ring [4, 18]. After administration, bendamustine is rapidly and irreversibly distributed and broken down via multiple pathways, primarily hydrolysis, with the cytochrome P450 (CYP) 1A2 oxidative pathway playing a minor role in its metabolism. Rituximab binds specifically to the CD20 antigen, a hydrophobic transmembrane protein [8], and is metabolized to peptides and amino acids that can be recycled in the body or excreted in the urine [19]. Oxidative metabolizing enzymes, such as CYPs, are not believed to be involved in rituximab elimination [20].

Biologics are typically unable to affect the pharmacokinetics of small molecules through direct effects, such as those pertaining to the induction or inhibition of CYP enzymes [19], but do have the potential to affect the disposition of small molecules through indirect effects, produced via alteration of the levels of cytokines or cytokine modulators that can affect CYP enzyme activity [20–24]. A biologic is unlikely to have an indirect effect on the pharmacokinetics of bendamustine, however, for two reasons: (1) Bendamustine is primarily metabolized via rapid hydrolysis and (2) The only CYP enzyme known to metabolize bendamustine plays only a minor role in its metabolic elimination. Metabolism of biologics can be specific (i.e., breakdown can occur via binding to a specific receptor) or nonspecific (i.e., breakdown can occur via hydrolysis by a protease). Small molecules can interfere with the metabolism of biologics by affecting the protease-mediated hydrolytic pathways [25]. Data are scarce on the pharmacokinetics of rituximab when administered as a component of different regimens [26] and on factors affecting individual exposure [20, 27].

Given clinical trial data demonstrating the benefits of bendamustine–rituximab combination therapy in patients with lymphoid malignancies, an investigation of the potential for drug–drug interactions is warranted. The purpose of the current analysis was to determine whether there is evidence of a drug–drug interaction between bendamustine and rituximab. The analysis dataset was based on data from two studies and the published rituximab literature. One of the studies is a multidose, multicenter, open-label, phase III study in adults with advanced indolent NHL or mantle cell lymphoma. The other study is a multicenter, open-label, single-agent, phase III investigation of the safety, efficacy, and pharmacokinetic profile of bendamustine (without concomitant rituximab) in adults with indolent NHL who were refractory to rituximab, which served as the basis for the bendamustine population pharmacokinetic model [18, 28].

## Methods

### Treatment regimens in the bendamustine–rituximab combination and bendamustine monotherapy studies

In the bendamustine–rituximab combination study, rituximab (375 mg/m^2^) was administered as an intravenous infusion on day 1 of each 28-day cycle for 6–8 treatment cycles; bendamustine (90 mg/m^2^) was administered as a 30-min intravenous infusion following completion of the rituximab infusion on day 1 of each cycle and was repeated on day 2 of each cycle. In the monotherapy study, bendamustine (120 mg/m^2^) was administered as a 60-min intravenous infusion on days 1 and 2 of 6 consecutive 21-day treatment cycles.

### Population pharmacokinetic model for bendamustine monotherapy

Previously, a population pharmacokinetic model for bendamustine (without concomitant rituximab) was developed using data from a phase III study in adults with indolent NHL who were refractory to rituximab [18, 28]. The data in this bendamustine monotherapy study included 100 patients. The patient population was primarily male (65 %) and Caucasian (88 %), with median body surface area of 2.0 m^2^ (range, 1.3–2.7 m^2^). Patient demographics and characteristics were generally similar to those in the bendamustine–rituximab combination study (Table 1). A subset of 80 patients were included in the model development dataset: 78 patients with bendamustine concentrations (12 had a complete pharmacokinetic profile collected on day 1 of cycle 1, and 66 had sparse pharmacokinetic samplings performed at predefined sample times) and two patients with metabolite concentrations only. Pharmacokinetic samples from the other 22 patients were excluded because they were not assayed within the validated stability period.

**Table 1:** Summary of patient characteristics

Patient characteristic	Bendamustine–rituximab combination (*n* = 49)	Bendamustine monotherapy [18] (*n* = 80)
Age (years)		
Median	64	57.5
Minimum, maximum	37, 84	31, 84
Body surface area (m^2^)		
Median	2.00	2.0
Minimum, maximum	1.4, 2.6	1.3, 2.7
Sex, *n* (%)		
Male	31 (63.3)	50 (62.5)
Female	18 (36.7)	30 (37.5)
Race, *n* (%)		
Caucasian	45 (91.8)	71 (88.8)
Black	–	5 (6.3)
Asian	1 (2.0)	1 (1.3)
Hispanic	–	1 (1.3)
Other	3 (6.1)	2 (2.5)

Following administration of bendamustine, the decline from peak plasma concentration occurred in a triphasic manner: the curve was characterized by a very rapid distribution phase, an intermediate phase, and a slower terminal phase. The population pharmacokinetic model that best described the bendamustine data was a 3-compartment, open model with zero-order input and first-order elimination [18]. The fixed effect parameter estimates were 31.7 L/h for clearance, 14.1 L for central volume of distribution (*V*_c_), 0.920 L for peripheral volume of distribution 1 (*V*_p1_), and 25.2 L for peripheral volume of distribution 2 (*V*_p2_). Bendamustine is rapidly eliminated from the plasma as shown by the estimated half-life of the first phase of decline in the concentration–time curve (*t*_1/2α_), second phase (*t*_1/2β_), and terminal phase (*t*_1/2γ_), which were 0.29, 0.7, and 110 h, respectively. The area under the concentration–time curve (AUC) of the terminal phase accounted for <1 % of the total AUC; therefore, the *t*_1/2_ of the *β* phase was considered to represent the bendamustine elimination half-life. Additionally, the predicted concentration at 12 h (*C*_12_) after the first dose was 0.272 ng/mL, and the ratio of *C*_12_ to *C*_max_ had a mean value of 0.00004.

### Creation of the bendamustine–rituximab combination analysis dataset

The analysis dataset was based on the following sources: (1) Data pertaining to bendamustine and rituximab in the bendamustine–rituximab combination therapy study, (2) data from the bendamustine monotherapy study, and (3) rituximab data from the published literature in which rituximab was administered without bendamustine. Bendamustine and rituximab samples with concentrations less than the lower limit of quantitation were excluded from analyses of the bendamustine–rituximab combination therapy study.

### Pharmacokinetic sampling data in the bendamustine–rituximab combination study

In the bendamustine–rituximab combination study, blood was drawn for measurement of plasma bendamustine concentrations for all patients (*n* = 49) during cycle 1 at the following times: on day 1 prior to infusion, at end of infusion (EOI), and at 0.25 and 0.5 h after EOI; and on day 2 prior to infusion, at EOI, and at 1 h after EOI. Following a protocol amendment, newly enrolled patients (*n* = 21) had additional blood samples collected on day 2 at 0.25, 0.5, 3, and 5 h after EOI. Plasma concentrations of bendamustine were determined by validated high-performance liquid chromatography with tandem mass spectrometry methodology; the lower limit of quantitation was 0.10 ng/mL.

Following the previously mentioned protocol amendment, blood was drawn from newly enrolled patients for measurement of serum rituximab concentrations during cycle 1 at the following times: in the absence of bendamustine (on day 1 prior to rituximab infusion and at EOI) and in the presence of bendamustine (on day 2 prior to the second bendamustine infusion, at any time during the visit on days 7 and 14, and prior to the rituximab infusion on day 28). Serum concentrations of rituximab were determined using a method based on the Meso Scale Discovery platform [data on file]; the quantitation range was 1.00–20.00 mcg/mL.

### Rituximab data from the published literature

The medical literature was searched for studies that included: (1) use of a rituximab infusion of 375 mg/m^2^; (2) pharmacokinetic sampling after the first dose of rituximab; (3) at least 1 sampling time that corresponded with the sampling times from the bendamustine–rituximab combination study (EOI, 24 h, 7, 14, or 28 days); and (4) summary statistics of concentrations or a graph of the observed concentrations versus time since dose.

Four publications met the above criteria and were used in a graphical analysis of rituximab [29–32]. The minimum, median, and maximum rituximab concentrations following the first dose reported in six populations from the four publications were entered and stored in a database for use in this analysis.

### Methods for evaluating the effect of rituximab on bendamustine

The potential for rituximab to affect systemic exposure of bendamustine was analyzed using observed data from the bendamustine–rituximab combination study (bendamustine administered in the presence of rituximab), and the above described population pharmacokinetic model developed using data collected in the absence of rituximab.

The analysis included a 3-step sequence. First, an evaluation using a procedure similar to the visual predictive check (VPC) [33] was performed to determine whether the population pharmacokinetic model developed using bendamustine concentrations in patients receiving monotherapy was applicable to bendamustine concentrations collected in patients receiving bendamustine–rituximab combination therapy. Second, boxplots were generated of model-predicted Bayesian clearance values in patients who received bendamustine in the presence of rituximab (combination therapy study) and in patients who received bendamustine in the absence of rituximab (monotherapy study). Third, a statistical test for differences in the log-transformed model-predicted Bayesian bendamustine clearance values in the presence and absence of rituximab was performed using the Wilcoxon’s signed rank test (*α* = 0.05).

### Methods for evaluating the effect of bendamustine on rituximab

The potential for bendamustine to affect systemic exposure of rituximab was evaluated using observed rituximab data from the bendamustine–rituximab combination study and rituximab data from the published literature in which rituximab was administered without bendamustine. The observed minimum, median, and maximum serum rituximab concentrations from the bendamustine–rituximab combination study and four publications describing data in six populations were plotted for scheduled pharmacokinetic sample times at the end of the rituximab infusion, 24 h post-infusion, and 7 days post-infusion.

### General statistical methods

All data preparations and presentations were performed using validated SAS software, version 9.2 (SAS Institute). Analyses conducted for the population pharmacokinetic model development and model simulations were conducted using NONMEM, version 6, level 2.0 (ICON Development Solutions).

## Results

### Description of bendamustine–rituximab combination pharmacokinetic data and the patient population

The source data in the bendamustine–rituximab combination study included 324 bendamustine pharmacokinetic sample records from 52 patients and 126 rituximab pharmacokinetic sample records from 21 patients. After exclusions and imputations, the final analysis dataset included 243 bendamustine sample records from 49 patients and 77 rituximab sample records from 19 patients. Fifty bendamustine samples and 34 rituximab samples were excluded because of concentrations less than the lower limit of quantitation.

The patient population in the bendamustine–rituximab combination study was primarily male (63 %) and Caucasian (92 %). Median body surface area was 2.0 m^2^ (range, 1.4–2.6 m^2^). In general, patient demographics and characteristics were similar to those in the bendamustine monotherapy study (Table 1).

### The effect of rituximab on bendamustine pharmacokinetics

The population pharmacokinetic model previously developed was used to simulate 500 bendamustine concentration–time profiles following administration of bendamustine monotherapy, assuming the same bendamustine dosing regimens and sampling times for patients as those in the bendamustine–rituximab combination study.

The median, 10th, and 90th percentiles of the simulated concentrations by scheduled sample time bins were calculated, and the median, 10th, and 90th percentiles of the observed concentrations in the bendamustine–rituximab combination study were also calculated. A plot was then generated to show the percentiles of the simulated monotherapy profiles overlaid with the observed concentrations of the combination therapy. Based upon VPC methods, the median, 10th, and 90th percentiles of the observed combination therapy and simulated monotherapy data should be similar.

**Fig. 1:**
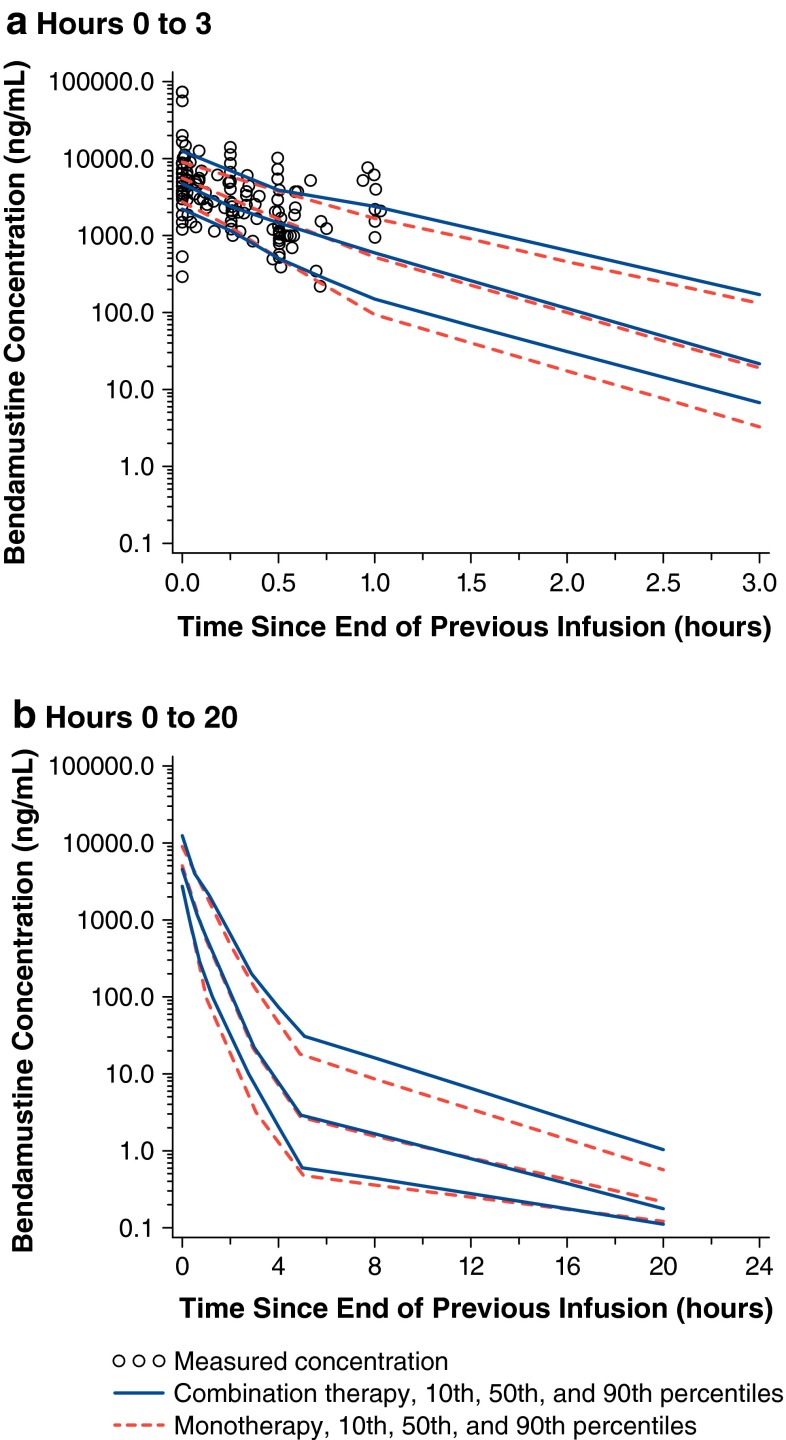
Observed bendamustine concentrations in the presence of rituximab overlaid with model-simulated bendamustine concentrations. Observed percentiles were calculated from actual data following coadministration of rituximab. Simulated percentiles were calculated from data simulated using the previously developed population pharmacokinetic model and doses, infusion durations, and sampling times for patients in the bendamustine–rituximab combination study

The VPC plot showed that the median, 10th, and 90th percentiles of the observed and simulated data were very similar (Fig. 1). Percentages of the observed bendamustine concentrations in patients receiving bendamustine–rituximab combination therapy that were below the 10th percentile and above the 90th percentile of the simulated concentrations for monotherapy were calculated. Overall, 26 samples (11 %) were below the 10th percentile, and 41 samples (17 %) were above the 90th percentile. Ideally, 10 % of the observed concentrations would be above the 90th percentile of the simulated concentrations, and 10 % of the observed concentrations would be below the 10th percentile of the simulated concentrations. The percentage of samples above the 90th percentile (i.e., 17 %) were higher than expected; however, many of the samples were obtained from one patient whose observed bendamustine concentrations were higher than those of the remaining population. Given the small number of subjects in the combination therapy study (*n* = 49) and the similarities in the median, 10th, and 90th percentiles, the findings suggest that the previously developed population pharmacokinetic model is applicable to the bendamustine–rituximab combination therapy data.

**Fig. 2:**
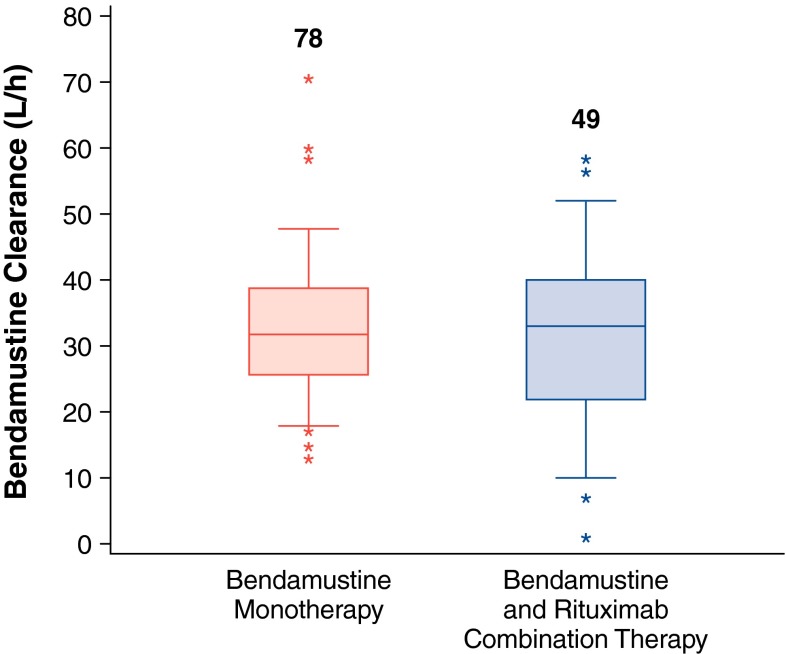
Bayesian estimates of bendamustine clearance with or without rituximab. *Boxes* are 25th, 50th, and 75th percentiles; *whiskers* are 5th and 95th percentiles. *Asterisks* are data points outside this range. The numbers above the *box* represent the number of patients

In addition to comparisons of concentration data, individual Bayesian estimates of bendamustine clearance in the presence and absence of rituximab were also analyzed (Table 2). The 25th–75th percentile range of Bayesian bendamustine clearance estimates indicated a slightly larger degree of variability between subjects; however, median bendamustine clearance values were similar at 32.9 versus 31.8 L/h in the presence and absence of rituximab, respectively (Fig. 2). The 2-sided Wilcoxon’s signed rank test of the log-transformed clearance values did not show a statistically significant difference between the two groups (*P* > 0.93), suggesting that rituximab does not affect the pharmacokinetics of bendamustine.

**Table 2:** Model-predicted Bayesian bendamustine clearance in the presence and absence of rituximab

Dosing regimen	Statistic	Clearance (L/h)	Log-transformed clearance^a^
Bendamustine–rituximab combination (*n* = 49)	Mean (SD)	32.1 (12.8)	3.33 (0.672)
	% CV	39.9 %	20.2 %
	Median	32.9	3.50
	Minimum, maximum	(0.9, 58.5)	(−0.097, 4.07)
Bendamustine monotherapy (*n* = 78)	Mean (SD)	33.0 (10.10)	3.45 (0.305)
	% CV	30.6 %	8.8 %
	Median	31.8	3.46
	Minimum, maximum	(13.1, 70.6)	(2.58, 4.26)

*% CV* percent coefficient of variation, *n* number of patients, *SD* standard deviation

^a^Wilcoxon’s signed rank test *P* > 0.93

### The effect of bendamustine on rituximab pharmacokinetics

In the four published studies used to investigate the effect of bendamustine on rituximab, the six comparator populations that reported rituximab concentrations included: (1) 22 patients with follicular lymphoma in complete or partial response after standard cyclophosphamide, doxorubicin, vincristine, and prednisone chemotherapy [29]; (2) 14 patients with various autoimmune disorders [29]; (3) 4 patients with amyloidosis [29]; (4) 137 patients with recurrent (up to four relapses) or refractory low-grade NHL [30]; (5) 10 patients with relapsed or resistant follicular or mantle cell lymphoma [31]; and (6) 7 patients with follicular NHL [32].

**Fig. 3:**
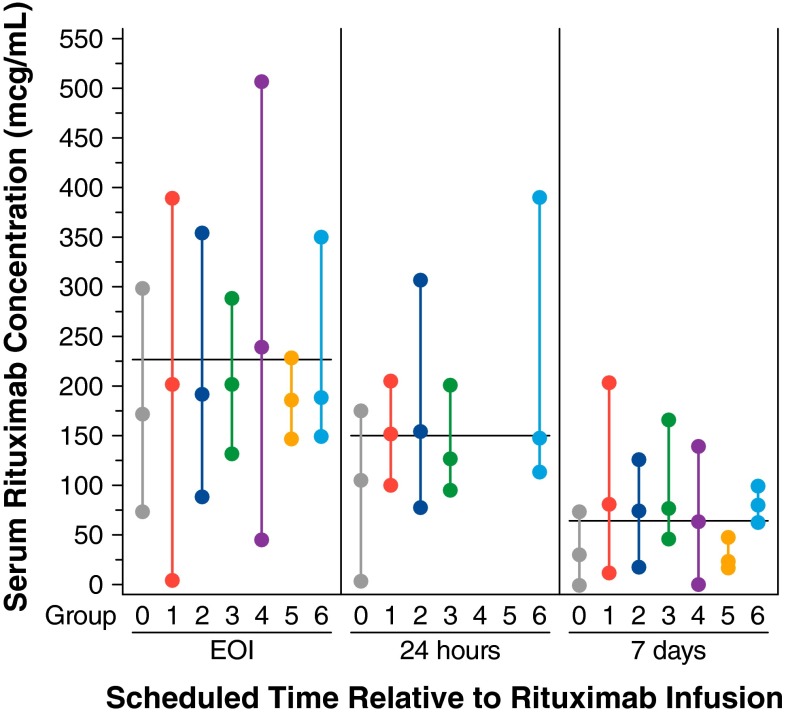
Median rituximab concentrations (min, max) over time from the bendamustine–rituximab combination study in the presence or absence of bendamustine. The lowest, middle, and highest symbols represent the minimum, median, and maximum of the observed rituximab concentrations, respectively. The *horizontal line* represents the weighted average of the median concentrations reported in literature only. *Note* For the bendamustine–rituximab combination study (Group 0), EOI concentrations were collected prior to bendamustine administration. The 24-h and 7-day concentrations were collected following bendamustine administration. For all other groups, rituximab was administered in the absence of bendamustine. *Group 0*: the bendamustine–rituximab combination study; *n* = 19; advanced indolent NHL or MCL. *Group 1*: NHL with low tumor burden [29]. *Group 2*: *n* = 14; autoimmune disorders [29]. *Group 3*: *n* = 4; amyloid light-chain amyloidosis [29]. *Group 4*: *n* = 137; recurrent low-grade or follicular NHL [30]. *Group 5*: *n* = 10; advanced follicular lymphoma and MCL [31]. *Group 6*: *n* = 7; follicular lymphoma [32]. *EOI* end of infusion, *MCL* mantle cell lymphoma, *NHL* non-Hodgkin lymphoma

In the absence of bendamustine, the median observed serum rituximab concentration at EOI in the bendamustine–rituximab combination study was about 54 mcg/mL (24 %) lower than the weighted average of the median concentrations reported in the six comparator populations (Fig. 3). Rituximab concentrations observed in the presence of bendamustine at 24 h and 7 days post-EOI were also lower than those reported in the literature (by about 45 mcg/mL [30 %] and about 35 mcg/mL [53 %]). The degree to which rituximab concentrations were lower was similar in the end-of-rituximab infusion sample (prior to bendamustine administration) and in the 24-h post-infusion sample (after the first infusion of bendamustine); however, at 7 days post-infusion, a larger difference in rituximab concentrations was noted. The general pattern of decline of rituximab concentrations observed in the bendamustine–rituximab combination study, from EOI to 24 h and 7 days, was similar to that seen in rituximab studies without bendamustine (six comparator groups), suggesting that bendamustine does not affect the rate of clearance of rituximab.

### Adverse event profile

In the bendamustine monotherapy study, the most common adverse events of all grades among the 100 treated patients included anemia (*n* = 94 [94 %]), thrombocytopenia (*n* = 88 [88 %]), neutropenia (83 [83 %]), nausea (77 [77 %]), infection (69 [69 %]), and fatigue (64 [64 %]) [28]. Reversible myelosuppression, gastrointestinal toxicity, and infection were the major toxicities associated with bendamustine [28]. In the bendamustine–rituximab combination study, the most common adverse events of all grades were nausea (*n* = 38 [72 %]), fatigue (*n* = 27 [51 %]), constipation (*n* = 22 [42 %]), neutropenia or infusion-related reaction (*n* = 19 [36 %] each), vomiting or diarrhea (*n* = 16 [30 %] each), pyrexia or insomnia (*n* = 12 [23 %] each), and thrombocytopenia, decreased appetite, or cough (*n* = 11 [21 %] each). Among adverse events associated with treatment, the most common were nausea (*n* = 32), fatigue (*n* = 20), neutropenia (*n* = 18), constipation or vomiting (*n* = 14 each), and thrombocytopenia or diarrhea (*n* = 11 each). Adverse events observed in both studies were generally consistent with the known adverse event profile of bendamustine [4, 28].

## Discussion

Few studies have been conducted primarily to investigate the metabolic mechanisms associated with drug–drug interactions between monoclonal antibodies and small molecules [11, 20]. Nonetheless, several potential mechanisms of drug interactions have been proposed. One hypothesis is that a drug interaction could be caused by modulated activity between the monoclonal antibody and Fcγ receptors on effector cells or by the simultaneous effect of the small molecule drug on Fcγ receptor expression [21]. For example, the downregulation of FcγRI on monocytes induced by methotrexate has been reported in patients with rheumatoid arthritis [21, 24]; such activity could potentially affect the action of a concomitantly administered monoclonal antibody. Another hypothesis is that a monoclonal antibody could affect the metabolism of a concomitantly administered small molecule drug via cytokine-induced CYP3A4 inhibition [22, 23]. Finally, the disposition of some small molecule drugs could also potentially be affected by changes in drug transport proteins due to treatment with a monoclonal antibody [20].

Although the pharmacokinetic characteristics of rituximab and bendamustine do not support a scientific rationale for a drug–drug interaction, the pharmacokinetics of coadministered rituximab and bendamustine have not been investigated previously, aside from a 2011 pilot study conducted in Japan. This small (*n* = 9) study suggested that rituximab does not affect the pharmacokinetic profile of bendamustine [34]. In the current analysis, data from the phase III bendamustine–rituximab combination study, in which bendamustine was coadministered with rituximab, and the previously developed population pharmacokinetic model based on data from the phase III bendamustine monotherapy study [18] were used to compare the pharmacokinetics of bendamustine in the presence and absence of rituximab. The analysis showed similar bendamustine concentration–time profiles as well as Bayesian bendamustine clearance estimates (with no statistically significant difference) in the presence and absence of rituximab.

Findings from published studies measuring the serum concentrations of rituximab (in the absence of bendamustine) at specific time points after infusion show a pattern that is the same as the one reported in the current bendamustine–rituximab combination study (i.e., similar pattern of decline for serum concentration of rituximab over time). Rituximab concentrations in the presence and absence of bendamustine in the current analysis were found to be consistently lower than those reported in the literature, with a similar difference in concentration at EOI of rituximab (prior to bendamustine administration) and 24 h post-infusion (following the first infusion of bendamustine), and a larger difference in concentration at 7 days post-infusion; however, all of the disparities could potentially be due to differences in the duration of the rituximab infusion or assay methodology/sensitivity.

The current analysis has several limitations, including the lack of a direct measurement of the effect of bendamustine on the pharmacokinetic profile of rituximab. In addition, since within-study comparisons were not possible, supplementation with cross-study comparisons or literature searches was necessary. The sparse sampling strategy used in the bendamustine–rituximab combination study also made it necessary to use a Bayesian prediction approach to generate individual predicted bendamustine clearance values. There are inherent constraints in the investigation of drug–drug interactions in patients with cancer; however, the methodology used in this analysis may provide a helpful tactic in this setting.

## Conclusions

Bendamustine in combination with rituximab has been reported to be an effective therapy in patients with lymphoid malignancies, yet data are lacking on the potential for pharmacokinetic interactions to occur between the two drugs. The current analysis, which used data from two studies and the published literature to determine whether there is evidence of a drug–drug interaction between bendamustine and rituximab, generated several key findings. First, the previously developed population pharmacokinetic model for bendamustine, which adequately described the concentration–time profile in adults following the administration of monotherapy, was also shown to adequately describe the concentration–time profile for bendamustine following the administration of combination therapy. Second, model-predicted bendamustine clearance was not statistically different when administered alone or in combination with rituximab, thereby suggesting that rituximab does not affect the pharmacokinetics of bendamustine. Finally, in the current analysis, the general pattern of decline in serum concentrations of rituximab after infusion mirrored that in published studies, although rituximab concentrations in the presence and absence of bendamustine were consistently lower than those reported in the literature. However, the degree of difference between the study and literature data suggest that the differing results were due to differences between studies in techniques rather than reflective of drug–drug interaction between bendamustine and concurrent treatment. Based on this analysis, therefore, neither bendamustine nor rituximab seems to affect the systemic exposure of the other drug when administered as combination therapy.
